# The Spermidine Synthase Gene as a Reporter of Transcription Inhibition in *Escherichia coli*

**DOI:** 10.3390/ijms27114829

**Published:** 2026-05-27

**Authors:** Anton R. Izzi, Alisa P. Chernyshova, Mikhail Y. Zhitlov, Alexander Yu. Rudenko, Ratislav M. Ozhiganov, Yury A. Ikhalaynen, Inna A. Volynkina, Lubov V. Dorofeeva, Vadim N. Tashlitsky, Igor A. Rodin, Lyudmila I. Evtushenko, Vera A. Alferova, Petr V. Sergiev, Olga A. Dontsova, Dmitrii A. Lukianov

**Affiliations:** 1 Center for Bio- and Medical Technologies, 121205 Moscow, Russia; antonizzi5@mail.ru (A.R.I.); abeidy@mail.ru (A.P.C.); vialabgroup@gmail.com (I.A.V.); petya@belozersky.msu.ru (P.V.S.); olga.a.dontsova@gmail.com (O.A.D.); 2 Faculty of Bioengineering and Bioinformatics, Lomonosov Moscow State University, 119234 Moscow, Russia; 3 Belozersky Institute of Physico-Chemical Biology, Lomonosov Moscow State University, 119234 Moscow, Russia; droplbox38@gmail.com (M.Y.Z.); rudriccc@gmail.com (A.Y.R.); lego-ratislav@yandex.ru (R.M.O.); alferovava@gmail.com (V.A.A.); 4 Shemyakin-Ovchinnikov Institute of Bioorganic Chemistry, Russian Academy of Sciences, 117997 Moscow, Russia; 5 Department of Chemistry, Lomonosov Moscow State University, 119234 Moscow, Russia; ikh.ya@yandex.ru (Y.A.I.); tashlitsky@belozersky.msu.ru (V.N.T.); igorrodin@yandex.ru (I.A.R.); 6 All-Russian Collection of Microorganisms (VKM), Pushchino Scientific Center for Biological Research, Russian Academy of Sciences, 142290 Pushchino, Russia; dekabr28@rambler.ru (L.V.D.); lie99@mail.ru (L.I.E.)

**Keywords:** antibiotics, mechanism of action, transcriptional inhibitors

## Abstract

Antimicrobial resistance is a major threat to modern society and healthcare, as it severely compromises the efficacy of standard antibiotic treatments. To meet the ever-increasing demand for novel antimicrobial drugs, it is crucial to develop new strategies for screening antimicrobial compounds and improve existing high-throughput techniques. Reporter systems that employ specific genetic markers are powerful tools not only for detecting antimicrobial activity of the substance being studied, but also for identifying the potential mechanism of its action. Among other metabolic pathways, RNA biosynthesis machinery is considered a promising molecular target as it remains underutilized in current antimicrobial therapy and therefore is rarely exposed to drug pressure. However, there is no suitable biomarker for identifying compounds that inhibit the transcription in Gram-negative bacteria. Combining bioinformatic search and RT-qPCR experimental validation, we have established the overexpression of the spermidine synthase gene (*speE*) as a biomarker associated with impaired transcription in *Escherichia coli*. Monitoring the expression level of *speE* in antibiotic-treated cells enables reliable detection of compounds that inhibit bacterial RNA-polymerase, such as rifampicin and fidaxomicin. Moreover, our screening system was successfully applied in practice to analyze chromatography fractions from fermentation broth of antibiotic producers, with compounds of the rifamycin family being identified as hits and isolated. The proposed method has the potential to be used in sequential screening procedures to reveal active antimicrobial compounds that inhibit bacterial transcription process, giving the world novel antimicrobials with minimal risk of resistance development.

## 1. Introduction

Antibiotics have become a powerful tool in the fight against infectious diseases, which were previously responsible for massive deaths due to the lack of adequate treatment methods or the insufficient effectiveness of existing treatments. Paul Ehrlich’s discovery of salvarsan, an effective antibacterial agent, was the result of many years of research [1]. It followed the discovery of the well-known antibiotic penicillin by Alexander Fleming during his experiments with *Penicillium notatum* [2]. The Waksman platform, which involves testing a variety of soil microorganisms on a plate covered with a layer of pathogenic bacteria, has led to the discovery of many antimicrobial agents during the golden age of antibiotics [3]. However, the antibiotic-resistant strains of pathogenic bacteria have become more prevalent. The formation of an antibiotic resistance crisis led to a decrease in the efficiency of known antibiotics due to the uncontrolled use of antimicrobial compounds in agriculture and pharmaceuticals [4].

A variety of strategies have been proposed to address this challenge. Multidrug therapeutic approaches are among the strategies that enable reducing the risk of resistance development without affecting the efficacy of treatment. By exploiting drug–drug interactions and utilizing collateral sensitivity, resistance-conferring mutations for one agent usually increase bacterial vulnerability to alternative agents. In addition, bacterial defense mechanisms, that are naturally present, might serve as another therapeutic tool [5]. For example, CRISPR-Cas nucleases are sequence-specific antimicrobials that target horizontally acquired genes that confer antibiotic resistance [6]. There are also potential alternatives to conventional antibiotics such as the use of probiotic microorganisms [7].

High-throughput screening based on whole-cell assays has revealed its weakness in efficient discovery of new antimicrobial agents nowadays. Combining the screening of natural and synthetic compounds with the simultaneous identification of their mechanism of action is considered the best strategy for discovering new antimicrobial agents. This leads to a reduction in research time and a substantial improvement in screening efficiency. Several reporter systems have been created that involve Gram-positive [8,9] and Gram-negative [10,11] bacteria, which enable to determine the mechanism of action of the tested antibiotic by activating the expression of a particular reporter gene. Most of those systems are aimed at screening antimicrobials that target protein synthesis or cause DNA damage, as well as reporter constructs that are capable of detecting quorum-sensing signaling molecules [12,13]. The genes employed as the basis for the development of reporter systems (biomarkers) are commonly those that react to the inhibition of various biosynthetic pathways [14,15]. Nevertheless, there are still no suitable markers that can efficiently detect antimicrobials affecting transcription in the *E. coli* system. Existing reporter systems based on luciferase assays do not provide enough specificity and cannot distinguish between transcription and translation inhibitors [16].

In this work, we present a specific *E. coli* model marker gene that indicates the impact of antimicrobials on transcription inhibition. We discovered it using an approach based on a combination of transcriptome analysis and experimental validation of potential marker genes, confirmed by quantitative PCR. Furthermore, the expression of this gene was assessed to screen column chromatography fractions for the presence of antimicrobial compounds that suppress bacterial transcription.

## 2. Results

### 2.1. Bioinformatic Search for Genes That Were Overexpressed After Rifampicin Treatment

To perform a screening system based on qPCR assay and with the aim to find antimicrobials that inhibit the transcription process in bacteria, it is necessary to discover the genes specifically overexpressing under the influence of antibiotics like rifampicin, which is a compound that inhibits bacterial RNA polymerase by binding to its *β*-subunit (RpoB), thereby affecting the transcription elongation stage [17]. For Gram-positive bacteria, including the *Bacillus subtilis* strain, a specific marker has been found—the *yppS* gene [9]. This gene has been shown to be upregulated upon treatment with RNA polymerase inhibitors, namely rifampicin and fidaxomicin. Therefore, its promoter was used as a part of a bioreporter assay adapted for soft-agar screening. However, the discovery of similar markers for Gram-negative bacteria has not yet been made. Due to this, an *E. coli* model has been chosen to solve this issue as there are extensive datasets of transcriptome data for this microorganism after exposure to several antibiotics, including rifampicin. A transcriptome dataset (accession number PRJNA889731) was chosen for this analysis, where in the *E. coli* K-12 strain MG1655 was exposed to three antibiotics with differing mechanisms of action—namely, rifampicin (50 µg/mL), novobiocin (100 µg/mL), and tetracycline (12.5 µg/mL). Therefore, the goal was to identify genes that are overexpressed when exposed to rifampicin but are downregulated or do not change their expression level when treated with other antibiotics.

Antibiotic treatment has resulted in significant changes in the *E. coli* transcriptome. Table 1 provides information on the properties of the transcriptome. The genes were considered differentially expressed at an adjusted *p*-value threshold <0.05 (using the Benjamini–Hochberg procedure [18]), as well as at a |log2FoldChange| threshold >0.58 to be considered as moderate, biologically meaningful changes [19,20]. An average of 2498 genes were differentially expressed under antibiotic treatment, accounting for 55.9% of the genome. The average number of genes upregulated and downregulated under antibiotic stress was roughly equal (1220 vs. 1278), and the percentage of differentially expressed genes did not differ significantly between the different treatments.

To identify genes that are specifically upregulated after rifampicin treatment, a log2FoldChange threshold of 2.37 was set with an adjusted *p*-value threshold of <0.05. In other treatments, these genes should have been significantly downregulated or not considered significant. This criterion led to the selection of eight genes, which are presented in Table 2. The products of the most of the selected genes are considered to be involved in different metabolic pathways, and only the *yiaG* gene product is involved in the transcription process. These genes were chosen to be confirmed further by qPCR on the *E. coli* K12 strain. Moreover, a *B. subtilis* marker *yppS* gene, which stands for phosphoenolpyruvate synthase, was also involved in the study, which is a *ppsA* gene in *E. coli*.

### 2.2. Analysis of the Expression Level of Potential Marker Genes by RT-qPCR

To confirm the possibility of using bioinformatically discovered genes as markers of transcription inhibition in experimental conditions, an *E. coli* K12 culture was treated with different antibiotics. Among these compounds were rifampicin as the main treatment, holomycin, ciprofloxacin, and erythromycin as antibiotic controls, as well as DMSO and deionized water as negative controls. Antibiotics concentration was selected according to the minimum inhibitory concentration. The MIC values of antibiotics used are given in Table S1. In total, 2x MICs were chosen as the middle variant that may affect gene expression and do not significantly reduce cell viability. After incubation for 30 and 60 min, total RNA was isolated and analyzed by RT-qPCR with primers for the genes of interest. In addition, a primer set for the *sulA* gene was used to test for ciprofloxacin treatment as it is a marker of SOS response in *E. coli* [21]. The minimum threshold for the change in gene expression at which a particular antibiotic treatment is considered to cause an effect was set at ≥1.5 with a *p*-value < 0.05. This threshold is often used to identify genes that are expressed differently [22,23,24].

**Figure 1:**
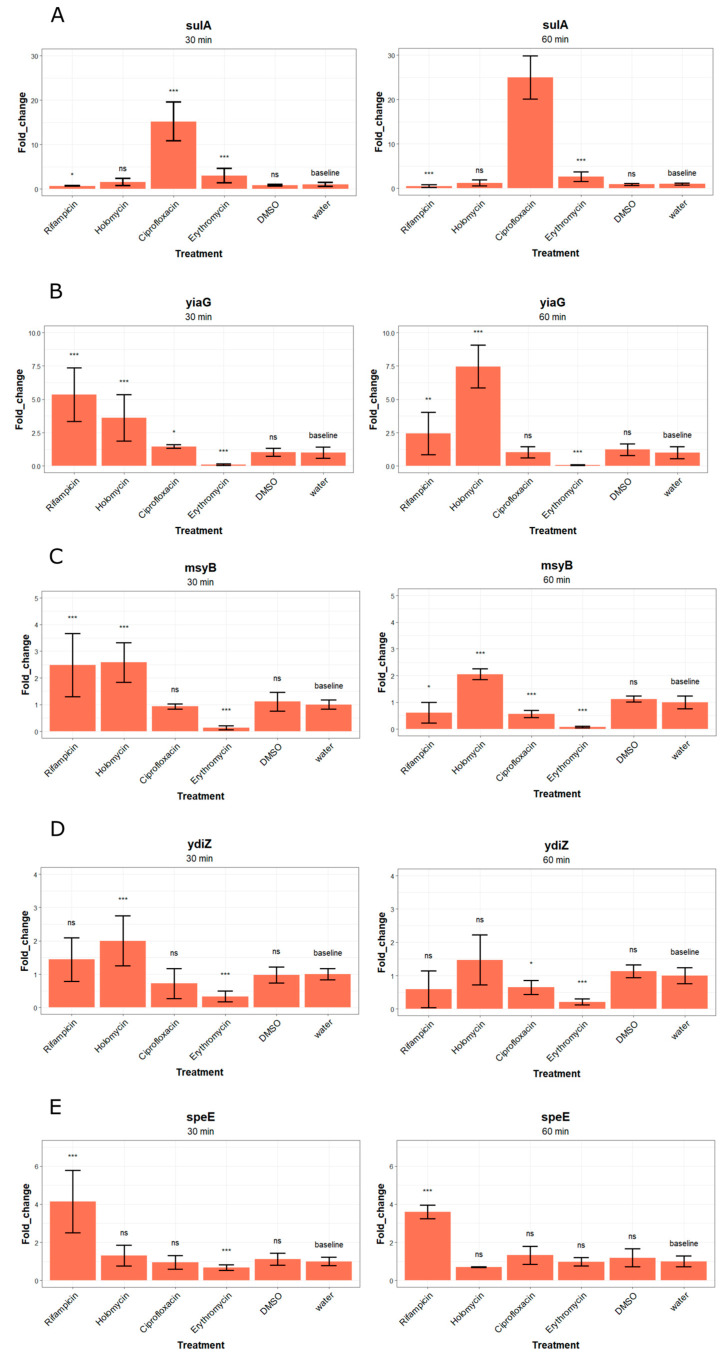
The expression values of *sulA* gene and potential marker genes measured by RT-qPCR after treatment of *E. coli* K12 strain with different antibiotics at 2x MICs (* *p* < 0.05; ** *p* < 0.01; *** *p* < 0.001; ns: not significant, by one-way robust ANOVA and mcppb20 post hoc test). (**A**) Expression values for *sulA* gene. (**B**) Expression values for *yiaG* gene. (**C**) Expression values for *msyB* gene. (**D**) Expression values for *ydiZ* gene. (**E**) Expression values for *speE* gene.

RT-qPCR results showed that the *sulA* gene under ciprofloxacin treatment demonstrated significant overexpression at 30 and 60 min of incubation (*p* < 0.001, one-way robust ANOVA test) (Figure 1A). The choice of non-standard robust ANOVA test is explained by large difference in dispersion between different treatments (max(s^2^)/min(s^2^) > 3–4 [25]), so the classical ANOVA test cannot be applied. The *yiaG* gene has demonstrated a relatively high expression level after 30 min of treatment with rifampicin (*p* < 0.001, one-way robust ANOVA test) (Figure 1B). This is confirmed by the data of bioinformatic analysis. Nevertheless, treatment with holomycin has also led to an increase in gene expression after 30 min incubation (*p* < 0.001, one-way robust ANOVA test). After 60 min, the yiaG gene demonstrated a decrease in expression level after rifampicin treatment (*p* < 0.01, one-way robust ANOVA test), while it increased after holomycin treatment (*p* < 0.001, one-way robust ANOVA test). Holomycin belongs to the group of antimicrobial natural products known as dithiolopyrrolones, which were previously thought to target RNA polymerase [26]. It also acts as a zinc ion chelator [27]. Holomycin treatment of *E. coli* results in inhibition of class II fructose bisphosphate aldolase but not RNA polymerase [28]. Therefore, the *yiaG* gene cannot be used as a reliable marker for bacterial transcription inhibition.

Similar results were observed for the *msyB* gene (Figure 1C), which demonstrated a significant (*p* < 0.001 at 30 min, one-way robust ANOVA test), but not a big enough overexpression level after treatment with rifampicin and holomycin. However, after 60 min of incubation, rifampicin treatment led to a significant decrease in the expression level of the gene (*p* < 0.05, one-way robust ANOVA test). In the case of holomycin treatment, both 30 and 60 min incubations resulted in significant overexpression level of *msyB* gene with almost the same fold change value (*p* < 0.001, one-way robust ANOVA test). The expression level of the *ydiZ* gene did not change significantly due to rifampicin treatment (*p* > 0.5, one-way robust ANOVA test) (Figure 1D). The expression levels of genes *aceB*, *aceA*, *luxS*, *ahr* and *ppsA* were generally not significantly different from the control (*p* > 0.5, one-way robust ANOVA test) for almost every treatment (Figure S1). All of the potential marker genes mentioned above, due to their demonstrated expression properties when treated with various antibiotics, cannot be used as indicators of transcription inhibition and therefore were excluded from further analysis.

The last of the tested genes, *speE* (Figure 1E), has demonstrated significant overexpression after treatment with rifampicin (*p* < 0.001 at 30 and 60 min, one-way robust ANOVA test), while almost all other treatments, including holomycin, have not significantly changed the expression level of the gene (*p* > 0.05 at 30 and 60 min, one-way robust ANOVA test). This spermidine synthase gene was chosen to be further confirmed as a potential marker of bacterial transcription stress.

### 2.3. Analysis of the Expression Level of speE Gene in E. coli lptD^mut^ Strain Model

**Figure 2:**
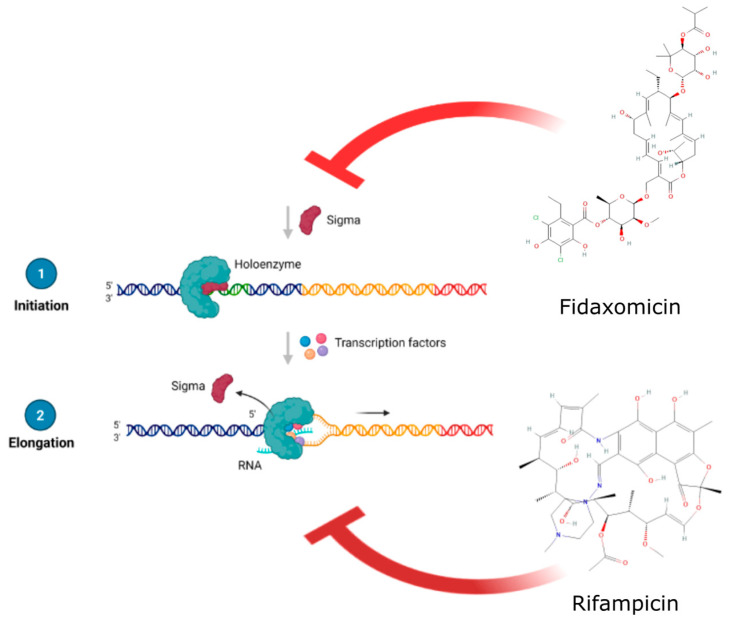
The structures of rifampicin and fidaxomicin and their schematic impact of their affect on different stages of bacterial transcription. Rifampicin acts as an inhibitor of transcription elongation via binding to the *β*-subunit deep within the DNA/RNA channel of bacterial RNA polymerase; at the same time, fidaxomicn is mostly an initiation step inhibitor.

At this moment, there are not many antimicrobial compounds, particularly in readily available forms, that can directly or indirectly disrupt bacterial transcription and simultaneously have an effect on Gram-negative bacteria. In the next steps of the experiments, *E. coli lptD^mu^*^t^ [29] was used. It has a deletion in the LPS-assembly protein LptD gene, and is characterized by increased susceptibility to antimicrobials. In some cases, it can be affected by compounds that primarily work on Gram-positive microorganisms. The next qPCR experiment was performed to analyze *speE* gene expression after antibiotic treatments using *E. coli lptD^mut^* as a model microorganism, with fidaxomicin as another positive control that affects the initiation of transcription process, thereby demonstrating distinct from the rifampicin mechanism of action (Figure 2) [30,31]. According to an agar plate test, fidaxomicin impeded the growth of the chosen strain (Figure S2), but the inhibition zone was significantly smaller than that of rifampicin. The weaker effect of fidaxomicin was also demonstrated by MIC values (31.25 µg/mL for fidaxomicin compared to 0.02 µg/mL for rifampicin). The test was carried out similarly to that for the K12 strain, except that holomycin was replaced by fidaxomicin, and antibiotics were tested at 5x MIC and 2x MICs.

**Figure 3:**
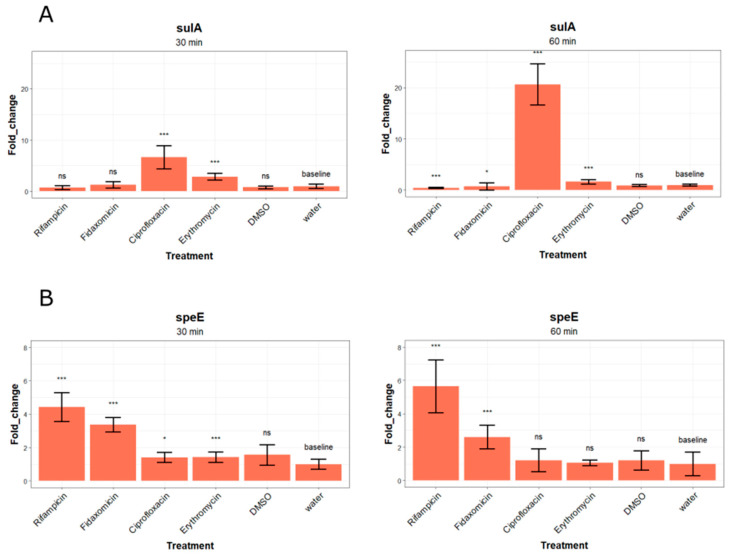
The expression values of *sulA* and *speE* genes measured by RT-qPCR after treatment of *E. coli lptD^mut^* strain with different antibiotics at 2x MICs (* *p* < 0.05; *** *p* < 0.001; ns: not significant, by one-way robust ANOVA and mcppb20 post hoc test). (**A**) Expression values for *sulA* gene. (**B**) Expression values for *speE* gene.

The *sulA* gene, after treatment with test antibiotics at 2x MICs, demonstrated almost the same expression profile as in the case of K12 cells. Ciprofloxacin treatment results in a significant increase in overexpression (*p* < 0.001 at 30 and 60 min, one-way robust ANOVA test) (Figure 3A). The wild-type strain had slightly different average expression values in this case. It can be explained by the specificity of the response to antibiotic stress in the susceptible-to-antimicrobials *E. coli lptD^mut^* strain.

The potential marker gene *speE* was significantly overexpressed after cells were treated with rifampicin (*p* < 0.001 at 30 and 60 min intervals, one-way robust ANOVA test) (Figure 3B), which supports the previous test. The same was observed for fidaxomicin treatment. The expression level of the *speE* gene also increased significantly when treated with this antibiotic (*p* < 0.001 at 30 and 60 min, one-way robust ANOVA test). Despite the treatment of ciprofloxain and erythromycin, significant differences in expression levels were found with the water control treatment (*p* < 0.05 and *p* < 0.001 respectively at 30 min, one-way robust ANOVA test). The mean expression levels of the *speE* gene after those treatments have not reached the minimum threshold required to be considered sufficient. Despite this, the *speE* gene’s expression level slightly increased after 60 min of rifampicin treatment, whereas it decreased after fidaxomicin treatment at the same incubation time. This might be attributed to the mechanism of action of fidaxomicin and the lack of susceptibility to it of the *lptD^mut^* strain in contrast to Gram-positive bacteria [32].

Incubation of *lptD^mu^*^t^ strain with 5x MIC antibiotic concentrations for 30 min led to a significant increase in the expression level of *speE* after rifampicin and fidaxomicin treatments (*p* < 0.001, one-way robust ANOVA test) (Figure S3). Nevertheless, the expression values for both treatments were noticeably lower than those of the treatment with a lower concentration. It can be explained with a significant increase in RNA synthesis inhibition, which to some extent neutralized the effect of overexpression of the marker gene. A large standard deviation in the case of DMSO treatment might also indicate additional influence of the vehicle as a stress factor in such concentration, despite its effect not being significant (*p* > 0.05, one-way robust ANOVA test). What is more, such higher antibiotic concentrations may disrupt the observed effect of treatment on detected gene expression levels due to its impact on bacterial viability, whereas 2x MIC may indeed have a minor effect on cell amount; this also needs the introduction of post-treatment normalization of purified RNA amount on cell viability, which makes the screening procedure more complicated. Therefore, for further screening procedures, the 2x MIC antimicrobial concentration and incubation time of 30 min were selected as the most suitable conditions.

### 2.4. Antimicrobial-Producing Strains’ Column Chromatography Fractions Screening

**Figure 4:**
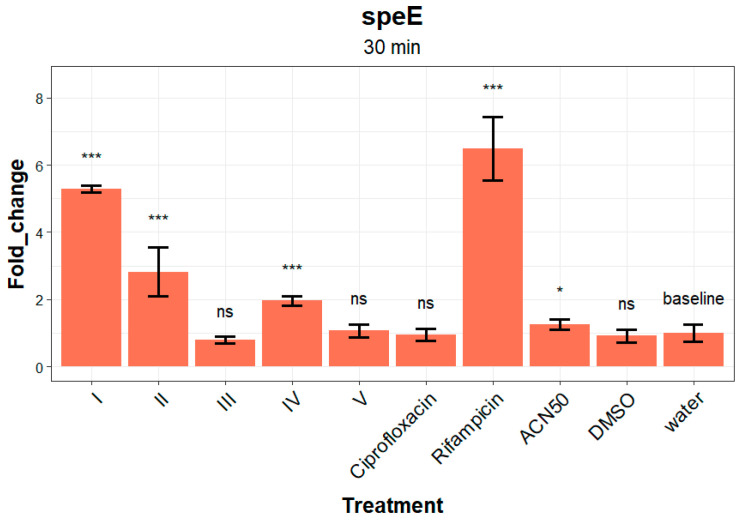
Screening of 5 column chromatography fractions from antimicrobial-producing strains (I, II, III, IV, V) using RT-qPCR method with *speE* gene as a marker (* *p* < 0.05; *** *p* < 0.001; ns: not significant, by one-way robust ANOVA and mcppb20 post hoc test).

To test the developed bacterial transcription stress screening system based on RT-qPCR procedure with primers specific for the *speE* gene, five samples were tested against *lptD^mut^* culture media. This media was obtained as a result of microorganism cultivation and was concentrated on column chromatography. Fractions with additional controls (rifampicin, ciprofloxacin, MeCN 50%, and DMSO) were used to treat *E. coli lptD^mut^* strain cultures. The in vitro agar plate test (Figure S4) showed that the fractions had a strong effect on the strain. The concentration of the active compound in the given fractions could not be measured because the concentration of antibiotics is unknown. The MIC value will be replaced with the concept of minimum inhibitory dilution (MID), which will be measured using a procedure that is similar to the calculation of MIC. As in previous treatments, column chromatography fractions were used 2x MID with 30 min of incubation. The results have shown a significant increase in *speE* gene expression after treatment with rifampicin, as well as in samples I, II, and IV (*p* < 0.001, one-way robust ANOVA test) (Figure 4).

The *speE* gene fold change values seen in sample I are similar to those observed in the control treatment with rifampicin, suggesting that sample I may contain a compound that can inhibit the transcription process in bacteria.

**Figure 5:**
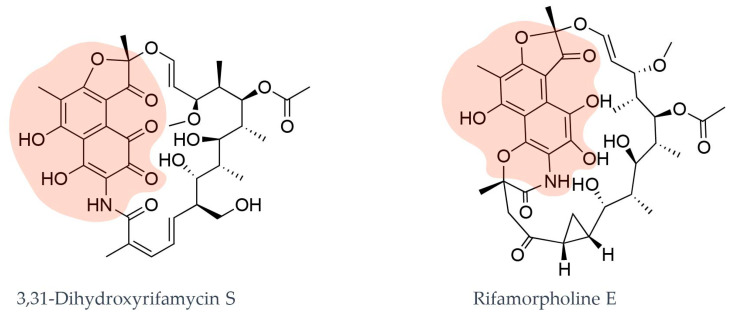
Chemical structures of 3,31-dihydroxyrifamycin S and rifamorpholine E. The fragment at *m*/*z* 273, characteristic of the naphthofuran moiety common to rifamycins, is highlighted.

To characterize this complex mixture, sample I (*Streptomyces* sp. VKM Ac-3162) was further separated by reverse-phase HPLC (Figure S5). The collected fractions were assessed for antibacterial activity, and a single active peak (retention time 12.8 min) was isolated for LC-MS analysis. HR-LCMS analysis of the active fraction revealed an ion at *m*/*z* 728.2602, corresponding to the [M+H]^+^ adduct, and an ion at *m*/*z* 726.2764, corresponding to the [M-H]^−^ adduct. These ions are consistent with the molecular formula C_37_H_45_NO_14_ (calculated mass 727.2836, Δ0.6 ppm), which matches both 3,31-dihydroxyrifamycin S and rifamorpholine E (Figure 5)—members of the rifamycin family. MS fragmentation in negative mode (Figure S6) showed a fragment at *m*/*z* 273, characteristic of the naphthofuran moiety common to rifamycins [33,34,35]. UV spectral data (Figure S5) further supports this assignment. Based on these data, together with the proposed RNA polymerase inhibitory mechanism known for rifamycins [36] and demonstrated using the *E. coli* BW25113 *lptD^mut^* reporter (Figure S4), the active compound can be confidently assigned to the rifamycin family. Given the biosynthetic richness of the rifamycin family [37], the studied strain is expected to produce multiple congeners of this class. This assignment confirms the correct operation of our screening system based on quantitative PCR, designed to search for compounds that disrupt the transcription process in bacteria.

## 3. Discussion

Bacterial resistance to antimicrobial substances is becoming an increasingly threatening global issue with harmful consequences if nothing is done about it. One of the main approaches to solving the problem is screening new potential antibiotics from synthetic molecule libraries and natural sources. The use of reporter systems significantly improves the procedure as they provide information about the potential mechanisms of action of certain antibiotics. It can be applied to standard model organisms (including *E. coli* and *B. subtilis*) as well as known pathogenic species [38,39,40]. Most of these reporter systems allow the identification of antibiotics that act on relatively common targets, such as ribosomes and DNA. However, there are several examples of systems that can detect antibiotics with specific targets [12,41,42].

A suitable target for screening and identification of new antimicrobials could be bacterial RNA polymerase. Antibiotics that target this enzyme are not widely used in clinical practice, and therefore there are relatively few pathogenic strains that have developed resistance to them. A reporter system aimed to identify transcription stressors via agar plate test has been developed for the *B. subtilis* model. The system consists of a promoter of the *yppS* gene linked to the reporter β-galactosidase gene, which showed stable induction after treatment with rifampicin and fidaxomicin [9]. Another *B. subtilis* high-throughput screening system for RNA synthesis stress was developed using the *yvgS* gene promoter fused to the firefly luciferase reporter gene [43]. However, there is no evidence that reporter constructs based on specific promoters can detect transcription inhibition of antibiotics specifically in Gram-negative bacteria.

In the present study, bioinformatic analysis of transcriptomic data from *E. coli* K12 treated with three different antibiotics—rifampicin, tetracycline, and novobiocin—revealed a set of genes whose expression levels were significantly elevated exclusively following rifampicin treatment. However, further analysis by quantitative PCR (qPCR) using an additional control antibiotic, holomycin, enabled the exclusion of the vast majority of these candidate genes, with the exception of the spermidine synthase gene (*speE*), which showed a marked and selective increase in expression only after rifampicin exposure. A subsequent qPCR experiment involving treatment of the *E. coli* lptD mutant strain with RNA polymerase inhibitors and the antibiotic fidaxomicin (as a positive control) demonstrated that *speE* can serve as a genetic marker for transcription inhibition stress. Moreover, there was no available transcriptomic data that compared *E. coli* rifampicin treatment with another set of antibiotics except translation and DNA stressors. Nevertheless, we have tried to analyze separate treatments of *E. coli* with ampicillin (PRJNA156979) and imipenem (PRJNA910221) treatments as cell wall synthesis inhibitors and colistin (PRJNA671752) treatment as a membrane disruptor; the results have shown no significant increase in *speE* gene expression level in all cases (Table S3). Finally, the proposed screening system based on RT-qPCR proved effective in analyzing culture fluids and chromatography fractions for the presence of RNA synthesis inhibitors, allowing the identification and isolation of rifamycin family compounds from column chromatography samples.

The relationship between transcription stress in *E. coli* and the specific overexpression of the spermidine synthase gene poses an important question. It is known that polyamines play a crucial role in maintaining bacterial cell homeostasis, as they are necessary for normal cell growth [44]. Additionally, they are crucial in the formation of biofilm [45] and play a role in regulating virulent factors in pathogenic bacteria [46]. Understanding the function of polyamines in bacterial cells may shed light on the possible explanations why the spermidine synthase gene was overexpressed when *E. coli* cells were exposed to transcription-disrupting compounds. Most spermidine in bacterial cell (1.4 mol) exists as a complex with RNA [47], and it can stimulate protein synthesis via decreasing Mg^2+^ concentration [48], as well as the synthesis of the products of the genes *rpoE* and *stpA* that are linked with transcription are stimulated by spermidine analogue called homospermidine [49]. So, it can be suggested that the increase in spermidine synthase gene expression after moderate transcription disruption with the acceleration of endogenous spermidine content may contribute to the compensation of the RNA synthesis inhibition via enhancing of transcription factor synthesis as well as accumulation of sigma factor associated with stress response. Interestingly, according to transcriptomic data the expression level of *rpoE* gene has not changed significantly (*p* > 0.05) (Table S2). Moreover, there is some experimental evidence in the literature about the possibility of polyamines to interact with free nucleotides [50,51], as well as spermidine also being capable of binding to ATP [47]; with the fact that some excess of nucleotide tri-phosphates may arise during transcription inhibition, it is possible that an increase in *speE* gene expression may lead to an increase in spermidine levels in the cell, which will bind to these excess nucleotides.

## 4. Materials and Methods

### 4.1. Bacterial Strains and Culture Conditions

In this work *E. coli* K12 and *E. coli* BW25113 *lptD^mut^* strains were used. Both strains were aerobically cultured in lysogeny broth (LB) medium at 37 °C overnight with constant shaking (200 rpm, 14 h). For antibiotic and MIC tests overnight, cultures were diluted 1:100 in fresh LB medium and incubated at 37 °C with constant shaking until OD600 reached 0.6. The antibacterial activity of the fractions was assessed using the reporter system *E. coli lptD^mut^* pDualrep2.1 [52]. *E. coli* BW25113 *lptD^mut^* carries a 69-nucleotide deletion in the *lptD* gene, which results in increased cell membrane permeability and consequently higher susceptibility to toxic compounds. The plasmid pDualRep2.1 drives expression of the red fluorescent protein TurboRFP upon activation of the SOS response, and the far red fluorescent protein Katushka2S upon disruption of translation.

For antibiotic tests, 5 mL of bacterial cultures were treated with 2x or 5x minimal inhibitory concentration (MIC) of antibiotic solutions or column chromatography fractions, as well as solvents of the corresponding antibiotics (DMSO, 50% MeCN) and deionized water as negative control. Treated cultures were incubated at 37 °C with constant shaking (200 rpm) for 30 and 60 min and then centrifuged (5700× *g*, 2 min) for collecting cell pellets; after removing the supernatant, the pellets were centrifuged again for 15 s to remove residual media, and after that they were frozen in liquid nitrogen and stored at −80 °C for further analysis.

### 4.2. Determination of Minimal Inhibitory Concentration (MIC)

Bacterial cultures with OD600 0.6 were diluted 1:1000 in LB medium and loaded into a 96-well plate in a volume of 100 µL, with an initial volume of 200 µL prior to serial dilution, as it was described here [53]. Stock solutions of antibiotics were added to the initial wells at appropriate concentrations (rifampicin—25 mg/mL, holomycin—25 mg/mL, ciprofloxacin—100 µg/mL, erythromycin—50 mg/mL for *E. coli* K12 strain, and rifampicin—100 µg/mL, fidaxomicin—50 mg/mL, ciprofloxacin—20 µg/mL, erythromycin—5 mg/mL in the case of the *E. coli lptD^mut^* strain). When column chromatography fractions were tested, 10 and 50 µL of each fraction were added to the 190 and 150 µL of diluted bacterial culture in initial wells respectively. In other wells diluted inoculated LB-culture media were added without antibiotics or column chromatography fractions, while the rest were left with LB media only as additional controls of sterility. A two-fold serial dilution was then carried out, with gentle mixing in each row. Afterwards those plates were incubated at 37 °C with shaking at 500 rpm in a plate thermo shaker (MB100-4A, Allsheng, Hangzhou, China), overnight. Cell growth was measured at 590 nm using a microplate reader (VICTOR X5Light Plate Reader, PerkinElmer, Shelton, CT, USA).

### 4.3. Data Analysis and Bioinformatics

For searching of genes, specifically overexpressing after rifampicin treatment, transcriptomic data of *E. coli* str. K12 substr. MG1655 control and antibiotic-treated cultures with accession number PRJNA889731 were used. An enriched library dataset was downloaded, and reads were mapped on *E. coli* genome using Bowtie2 program [54]. SAMtools were used for transformation of mapped reads into binary format and sorting [55]. FeatureCounts v2.0 [56] program was used for quantification of gene expression levels according to the genomic annotation corresponding to the MG1655 strain; the *E. coli* database used for mapping was GCF_000005845.2. For calculation of differentially expressed genes DESeq program on the R 4.5 platform was used [57], and for each antibiotic vs. control pair the calculations were performed in triplicates.

### 4.4. RNA Extraction and Quantitative Real Time-PCR (qPCR)

The expression values of candidate genes were measured by RT-qPCR. For this purpose, bacterial cultures at OD600 0.6 were treated for 30 and 60 min with antibiotics with different mechanisms of action, including transcription inhibitors, followed by RNA isolation and cDNA synthesis. The differential expression was calculated with respect to expression in the control using the 2 to the power of the minus delta-delta cycle threshold (2^−ΔΔCT^) method, normalizing for 23S rRNA as an endogenous control and for deionized water as a treatment control.

RNA from *E. coli* cells was isolated using RNA Solo kit (Evrogen, Moscow, Russia) according to the manufacturer’s protocol, and the concentration was measured using Qubit RNA HS Assay Kit (Life Technologies, Thermo Fisher Scientific Inc., Waltham, MA, USA). cDNA was synthesized from 1 μg of RNA by using Magnus reverse transcriptase (Evrogen, Russia).

Real-time PCR was performed in triplicate with CFX96 Touch Real-Time PCR Detection System (Bio-Rad, Hercules, CA, USA) using 5X qPCRmix-HS SYBR (Evrogen, Russia). Primer sequences of tested genes are given in Table 3.

### 4.5. Isolation and Identification of Bioactive Compound

To obtain a sufficient amount of the active compound for detailed bioactivity studies, strains was cultured in four 750 mL Erlenmeyer flasks with 150 mL of liquid PDS+starch (g/L: Grated potato 200.0, Glucose 20.0, Starch 10.0; pH 6.5–7.0, sterilized by autoclaving at 111 °C and 0.5 atm for 20 min) at 28 °C with shaking (200 rpm) for 10 days. Culture liquids were separated from biomass by centrifugation at 20,000× *g* for 20 min (Centrifuge 5810 R, Rotor FA-45-6-30, Eppendorf, Hamburg, Germany).

One liter of the clarified supernatant was loaded onto a 30 mL cartridge containing 7 g of LPS-500-H polymer sorbent (divinylbenzene hydrophilic copolymer, pore size 50–1000 Å, 70 μm; Technosorbent, Moscow, Russia) at a flow rate of 15 mL/min using a peristaltic pump (Masterflex L/S Variable Speed Pump System, Masterflex, Gelsenkirchen, Germany). Sequential elution was performed with 15 mL portions of water–acetonitrile (MeCN) mixtures containing 0, 10, 20, 30, 40, 50, 75, and 100% MeCN. The biological activity of each fraction was assessed using the reporter *E. coli lptD^mut^* strain [58].

Active column chromatography fraction was analyzed by reverse-phase HPLC using Agilent 1260 Infinity II (Agilent Technologies, Santa Clara, CA, USA) equipped with UV-Vis 1260 Diode Array detector WR (Agilent Technologies, Santa Clara, CA, USA). Separation was performed on a Nucleodur C18 gravity column (260 mm × 4.6 mm, 5 μm, 110 Å; Machery-Nagel, Duren, Germany) with solvent A (10 mM NH_4_OAc) and solvent B (MeCN). The elution profile consisted of gradient 20–60% eluent B over 10 min and a 4 min column wash with 60% eluent B with flow rate 1 mL/min and detection on 260 nm (Figure S4). The collected fractions were assayed for antibacterial activity; the fraction containing the pure active compound was isolated and further subjected to LC-MS analysis.

Mass spectra were collected using a maXis II 4G ETD mass spectrometer (Bruker Daltonics, Bremen, Germany) and UltiMate 3000 chromatograph (Thermo Fisher Scientific, Waltham, MA, USA), equipped with an Acclaim RSLC 120 C18 column (2.1 × 100 mm, 2.2 μm, Thermo Fisher Scientific, Waltham, MA, USA). The spectrum registration mode and ESI mode were determined with a full scan from *m*/*z* 100 to 1500, and MS/MS was performed with the selection of the three most intense ions, with the dissociation type CID 10–40 eV and nitrogen collision gas. Raw data were collected and processed on Thermo Xcalibur Qual ver. 4.3.73.11.

The identity of the samples was confirmed by HPLC-HRMS/MS analysis using the Orbitrap Exploris 240 mass spectrometer (Thermo Fisher Scientific, Waltham, MA, USA) coupled with the Vanquish UHPLC system (Thermo Fisher Scientific, Waltham, MA, USA), equipped with a reversed-phase Acclaim™ 120 C18 (2.2 µm, 2.1 × 150 mm) column (Dionex, Sunnyvale, CA, USA). Eluent A was 0.1% formic acid in ultrapure water; eluent B was 0.1% formic acid in acetonitrile. Chromatographic separation was carried out at 45 °C in gradient elution mode at a flow rate of 0.4 mL/min. The gradient program was as follows: 0–3 min, 5% B; 3–17 min, 5–95% B; 17–21 min, 95% B; 21–22 min, 95–5% B. Injection volume was 10 µL and autosampler temperature was 4 °C. Mass spectra were acquired separately in both positive and negative ionization modes. Heated electrospray ionization (ESI) source parameters were as follows: ion source voltage—3500 V for positive ionization mode or 2500 V for negative ionization mode; sheath/auxiliary/sweep gas—50/10/1 arb; ion transfer temperature—325 °C; vaporizer temperature—350 °C. Mass spectra were recorded with a full scan from *m*/*z* 50 to 2000. The resolution was 30,000 (for both MS1 and MS2 spectra), the RF lens—70%. MS2 spectra were acquired in data-dependent acquisition (DDA) mode using dynamic exclusion-based precursor selection. Higher-energy collisional dissociation (HCD) fragmentation parameters were as follows: isolation window—*m*/*z* 1 with isolation offset turned off; normalized collision energy—15%, 30%, 45%, and 60%; normalized automatic gain control (AGC) target—200% with a maximum injection time parameter set to “auto”.

At the stage of preliminary experiments low-resolution mass spectra (LRMS) were recorded by HPLC-MS analysis using the EXPEC L-Chrom MS triple quadrupole system (Expec, Hangzhou, China) equipped with an electrospray ionization source (ESI).

### 4.6. Statistical Analysis

Each experiment was performed with three replicates. qPCR quantitative data are presented as the mean ± standard deviation (SD). qPCR assays were assessed using one-way robust ANOVA based on 20% trimmed means (WRS2 1.1-7 package, R 4.5). Post hoc comparisons were conducted using mcppb20 function (percentile bootstrap, 20% means trimming). All statistical calculations and diagramming were performed in the Rstudio program; the ggplot2 4.0.0 package was used for figure drawing. The results were deemed significant when *p* < 0.05.

## 5. Conclusions

The antibiotic resistance crisis requires searching for new strategies for the discovery and identification of new antibiotics, as well as improving current instruments, including reporter systems. Searching for markers that can identify transcription inhibitors of antimicrobials in Gram-negative bacteria can serve as a valuable aid in the fight against antimicrobial resistance. This group of antibiotics is not widely used in clinical practice. Here, we identified the spermidine synthase gene as a suitable biomarker for the detection of RNA synthesis stressors using RT-qPCR method. The system can also be applied in screening cultural fractions obtained from antimicrobial-producing microorganisms, which can simplify the further exploration of novel transcription inhibitor compounds. The tool provided has the potential to be used in screening procedures that aim to find new antimicrobials that can treat resistant pathogens.

## Figures and Tables

**Table 1:** Number of differentially expressed *E. coli* genes after treatment with 3 different antibiotics.

Antibiotic	No. of Upregulated Genes	No. of Downregulated Genes	No. of Differentially Expressed Genes (% of Genome)
Rifampicin	1250	1304	2554 (57.2)
Tetracycline	1259	1406	2665 (59.7)
Novobiocin	1153	1124	2277 (51.0)

**Table 2:** The chosen *E. coli* genes as potential markers for transcription inhibition with their log2FoldChange values and significance levels for different antibiotic treatments.

Gene ID	Name	Treatment					
		Rifampicin		Tetracycline		Novobiocin	
		log2FoldChange	*p*adj	log2FoldChange	*p*adj	log2FoldChange	*p*adj
b3555	putative DNA-binding transcriptional regulator YiaG	4.392	1.895 × 10^−48^	−1.527	7.261 × 10^−8^	0.141	6.65 × 10^−1^
b1051	acidic protein MsyB	3.971	9.075 × 10^−47^	−1.105	5.213 × 10^−6^	0.069	8.42 × 10^−1^
b1724	putative endoribonuclease YdiZ	3.435	4.192 × 10^−33^	−0.076	7.934 × 10^−1^	0.835	4.74 × 10^−3^
b4014	malate synthase A	3.251	4.931 × 10^−29^	−0.690	1.302 × 10^−7^	0.046	8.77 × 10^−1^
b4015	isocitrate lyase	3.007	6.987 × 10^−25^	−2.270	1.669 × 10^−48^	0.108	6.80 × 10^−1^
b2687	S-ribosylhomocysteine lyase	2.655	4.456 × 10^−28^	−1.223	1.458 × 10^−16^	0.091	6.98 × 10^−1^
b0121	spermidine synthase	2.566	2.859 × 10^−18^	−0.212	3.317 × 10^−1^	−1.174	1.25 × 10^−7^
b4269	NADPH-dependent aldehyde reductase Ahr	2.379	1.778 × 10^−16^	−0.752	2.014 × 10^−3^	−0.988	2.76 × 10^−4^

**Table 3:** Primer sequences used for qPCR.

Primer Name	Gene	Sequence (5′-3′)
YiaG_F	Putative DNA-binding transcriptional regulator YiaG	AAAgATCCAATgCATgAgCTgTTgAgC
YiaG_R		TTAgTTCggCACTTgAAggCTTCAC
MsyB_F	Acidic protein MsyB	CgCAACgCTTgAAgAAgCCAT
MsyB_R		ACCgCTAAgCATAggTAAACATTCACC
YdiZ_F	Putative endoribonuclease YdiZ	TACCTgACTCgCgACggTTTTTT
Ydiz_R		CCCAggTCgTgATggTgATTTC
AceB_F	Malate synthase A	gACgggCAAATTAACCTgCgT
AceB_R		gCCTgATAgTTgTggAAgAAATAgAgCg
AceA_F	Isocitrate lyase	CTATCCggCAAACTCggTgC
AceA_R		CTTCACTgACgCCAgCTgATCT
LuxS_F	S-ribosylhomocysteine lyase	TTCgggTggCgAAAACAATgAAC
LuxS_R		gCgTACCAATCAgACTCATATAAAAACCgg
Ahr_F	NADPH-dependent aldehyde reductase Ahr	AggATAAAggTTTgCAggTCggT
Ahr_R		CAgTgATATggTgCATCAACAgTgg
SpeE_F	Spermidine synthase	gATgATgACCCATgTTCCgCTACT
SpeE_R		gCCATCgTCgATCACCAgCTTAAA
PpsA_F	Phosphoenolpyruvate synthetase	CgCCAgATgATgCgAggT
Ppsa_R		CAgAAgATATgCCggACgCTT
SulA_F	Cell division inhibitor SulA	TTCTACTgTTgCCATTgTTAC
SulA_R		CCgATCACCACACTgTA
RT_23S_F	23S ribosomal RNA	gCgAAATTCCTTgTCgggTAA
RT_23S_R		ACTTCAAAgCCTCCCACCTATCC

## Data Availability

Transcriptomic data used in this work were earlier deposited at NCBI BioProject under the accession number PRJNA889731.

## Supplementary Materials

The following supporting information can be downloaded at: https://www.mdpi.com/article/10.3390/ijms27114829/s1.
