# Supplementary material for: The Spermidine Synthase Gene as a Reporter of Transcription Inhibition in Escherichia coli
Source: Int J Mol Sci. 2026 May 27;27(11):4829. doi: 10.3390/ijms27114829 (PMC13256715; doi:10.3390/ijms27114829)
Supplement: Supplementary file 1 [file ijms-27-04829-s001.zip › ijms-4296603-supplementary.pdf]

# Supplementary Materials

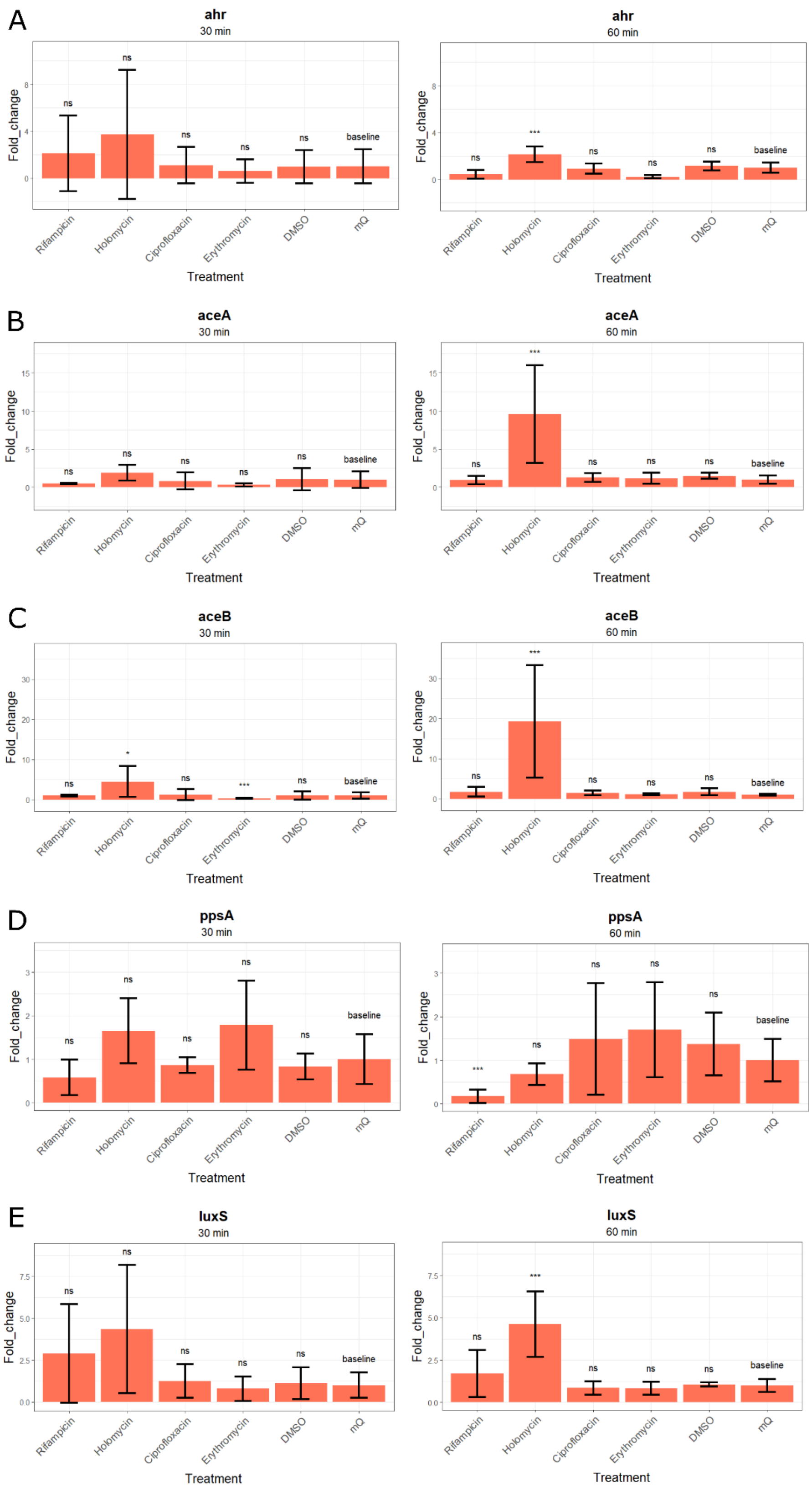


**Figure S1.** The expression values of *aceB, aceA, luxS, ahr* and *ppsA* potential marker genes measured by RT-qPCR after treatment of *E. coli* K12 strain with different antibiotics at 2x MIC

concentrations (*$p < 0.05$; **$p < 0.01$; ***$p < 0.001$, by one-way robust ANOVA and mcppb20 post hoc test).

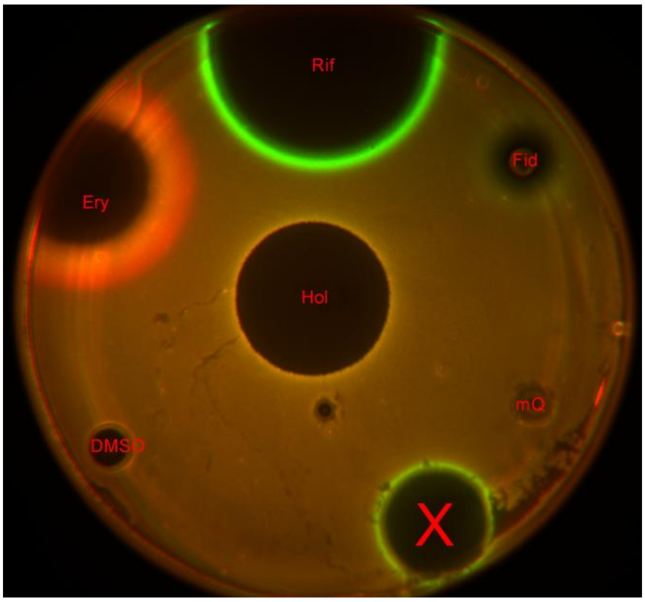


**Figure S2.** In vitro agar plate test with *E. coli lptD*$^{mut}$ pDualrep2.1 strain showing the activity of selected antibiotics. Erythromycin (Ery, 5 mg/ml) and rifampicin (Rif, 25 mg/ml) were used as positive controls. Holomycin (Hol) and Fidaxomicin (Fid) were tested at concentrations of 25 mg/ml and 50 mg/ml, respectively.

**Table S1.** Minimal inhibitory concentration of tested antibiotics on K12 and *lptD*$^{mut}$ *E. coli* strains.

| Antibiotic | MIC for *E. coli* K12, µg/ml | MIC for *E. coli lptD*$^{mut}$, µg/ml |
|---|---|---|
| Holomycin | 1 | not tested |
| Rifampicin | 8 | 0.02 |
| Fidaxomicin | not tested | 31.25 |
| Ciprofloxacin | 0.006 | 0.007 |
| Erythromycin | 31.25 | 0.78 |

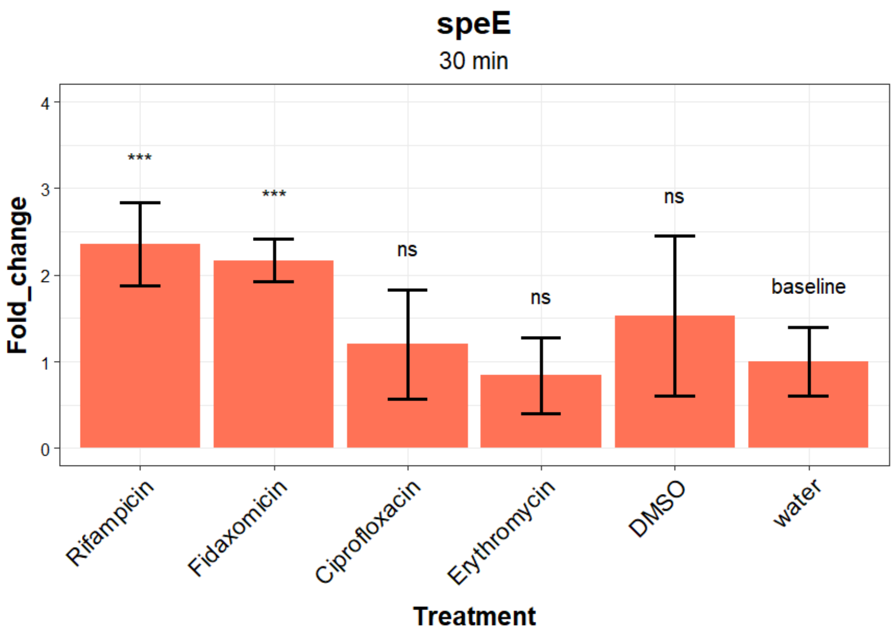


**Figure S3.** The expression values of the *speE* gene measured by RT-qPCR after treatment of *E. coli lptD*$^{mut}$ with different antibiotics at 5x MIC concentrations (*$p < 0.05$; **$p < 0.01$; ***$p < 0.001$, by one-way robust ANOVA and mcppb20 post hoc test). Effects of rifampicin and fidaxomicin treatments remain significant, but the expression values are less indicative than in the case of 2x MIC concentrations. Moreover, treatment with vehicle (DMSO) at the corresponding concentration has led to a vast increase in standard deviation.

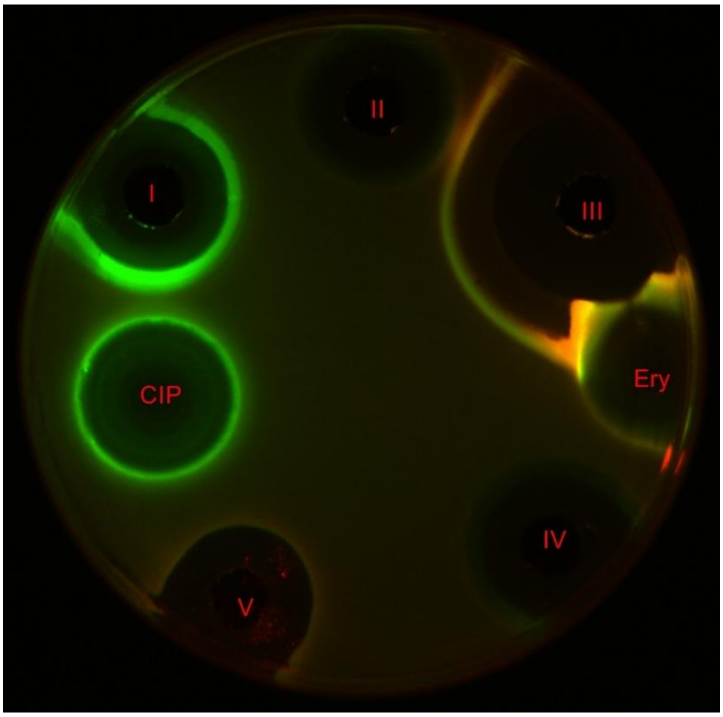


**Figure S4.** In vitro agar plate test with the *E. coli lptD*$^{mut}$ pDualrep2.1 strain for the activity of column chromatography fractions (I-V). Ciprofloxacin (CIP, 100 µg/ml) and Erythromycin (Ery, 5 mg/ml) were used as positive controls.

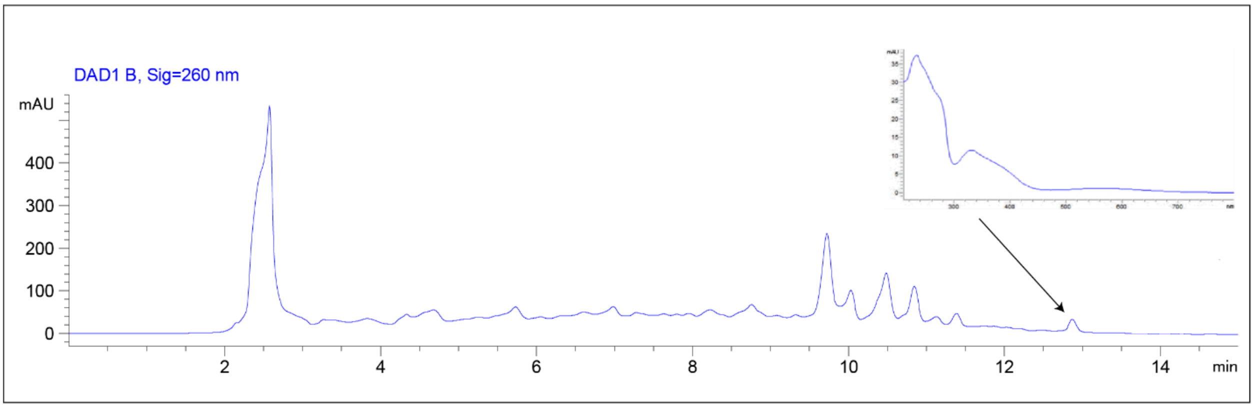


**Figure S5.** HPLC of the active fraction 44359-acn40 (column Nucleodur C18 gravity 260 x 4.6 mm, 5 µm); eluent solvent A – 10 mM $NH_4OAc$, pH 5, solvent B – MeCN; elution gradient: 20→60% solvent B over 10 min, followed by a 4 min column wash with 60% solvent B; flow rate 1 mL/min, detection at UV 260 nm. Active compound is eluted at 12.8 min. UV spectrum corresponding to the active peak is shown as an inset near the peak.

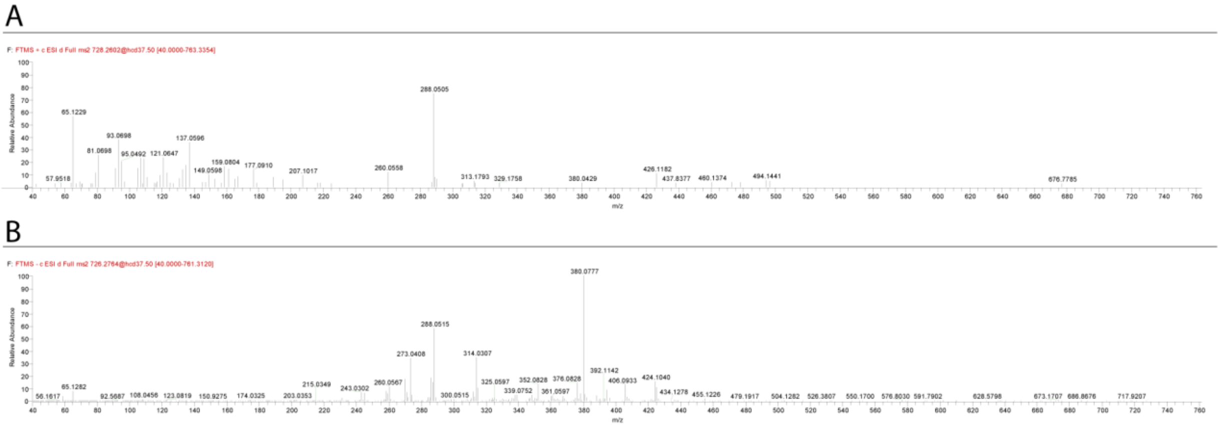


**Figure S6.** HCD mass spectra of precursor ions: (A) $[M+H]^+$ at m/z 728.2602 and (B) $[M-H]^-$ at m/z 726.2764.

**Table S2.** *E. coli* K12 *rpoE* gene log2FoldChange values and significance levels for different antibiotic treatments.

| Gene ID | Name | Treatment | | | | | |
|---|---|---|---|---|---|---|---|
| | | Rifampicin | | Tetracycline | | Novobiocin | |
| | | log2FoldChange | padj | log2FoldChange | padj | log2FoldChange | padj |
| b2573 | RNA polymerase sigma factor RpoE | 0.438 | 0.327 | -0.377 | 0.029 | -1.567 | 4.113E-15 |

**Table S3.** *E. coli speE* gene log2FoldChange values and significance levels for treatments with ampicillin (PRJNA156979), imipenem (PRJNA910221), and colistin (PRJNA671752).

| Gene ID | Name | Treatment | | | | | |
|---|---|---|---|---|---|---|---|
| | | Ampicillin | | Imipenem | | Colistin | |
| | | log2FoldChange | padj | log2FoldChange | padj | log2FoldChange | padj |
| b0121/ C1467_RS20535 | spermidine synthase | 0.296 | 0.4197 | 0.589 | 0.062 | -0.241 | 0.637 |
